# A Novel Fractional Accumulative Grey Model with GA-PSO Optimizer and Its Application

**DOI:** 10.3390/s23020636

**Published:** 2023-01-05

**Authors:** Ruixiao Huang, Xiaofeng Fu, Yifei Pu

**Affiliations:** 1 College of Computer Science, Sichuan University, Chengdu 610065, China; 2 Department of Security Application, Chengdu CETC 30, Chengdu 610093, China; 3 School of Automation, Nanjing University of Science and Technology, Nanjing 211103, China; 4 Chengdu Southwest Information Control Research Institute Co., Ltd., Chengdu 611630, China

**Keywords:** prediction of cyber security situation, fractional order, grey model, GA-PSO

## Abstract

The prediction of cyber security situation plays an important role in early warning against cyber security attacks. The first-order accumulative grey model has achieved remarkable results in many prediction scenarios. Since recent events have a greater impact on future decisions, new information should be given more weight. The disadvantage of first-order accumulative grey models is that with the first-order accumulative method, equal weight is given to the original data. In this paper, a fractional-order cumulative grey model (FAGM) is used to establish the prediction model, and an intelligent optimization algorithm known as particle swarm optimization (PSO) combined with a genetic algorithm (GA) is used to determine the optimal order. The model discussed in this paper is used for the prediction of Internet cyber security situations. The results of a comparison with the traditional grey model GM(1,1), the grey model GM(1,n), and the fractional discrete grey seasonal model FDGSM(1,1) show that our model is suitable for cases with insufficient data and irregular sample sizes, and the prediction accuracy and stability of the model are better than those of the other three models.

## 1. Introduction

More recently, there has been increasing interest in cyber security events with the rapid development of the Internet. The prediction of cyber security situations plays a crucial role in preventing the loss of security. There is a general paucity of studies focusing specifically on the prediction of security situations when the sample size is insufficient. To date, there has been little clear evidence that accuracy can be predicted when the sample size is insufficient, and samples are irregular.

Using the theory proposed by Deng [1], studies of grey models have traditionally used model systems to predict cyber security situations when the sample size is insufficient. GM(1,1) is the most popular and classic grey prediction model, and it is also the theoretical basis for the study of most grey prediction models. With the development of the grey system theory, many scholars have improved the traditional model to improve its prediction accuracy [2,3]. Ye studied the new energy consumption prediction model established by discrete GM(1,1) to solve China’s serious energy security and environmental pollution problems [4]. Lu combined heuristic fuzzy time series with an improved grey prediction model to improve the prediction accuracy of traditional GM(1,1) [5]. Zeng proposed a new structured grey Verhulst model by introducing a new nonhomogeneous exponential function and proved its performance [6]. Luo added a nonlinear time correction factor to improve the traditional GM(1,1) [7]. Building on the classical GM(1,1) model, scholars have extended the application of this grey model to discrete data [8] and developed the GM(1,n) model, which has also achieved good results.

However, grey models require samples to be exponentially distributed, which means that when fluctuation is high in the input samples, the prediction accuracy is reduced. The method that can effectively deal with this problem is called FAGM(1,1), which has improved prediction accuracy and flexibility in comparison with GM(1,1). Mao et al. [9] established a nonlinear fractional grey comprehensive model and predicted the urban road traffic flow. Shen et al. [10] proposed a weighted fractional-order cumulative grey prediction model based on the new information priority principle and a fractional accumulation generation operator. Liu et al. [11] proposed a fractional-order reverse accumulative discrete grey prediction model, which proved that the disturbance bound of the model solution is smaller than that of the traditional discrete grey model. Zeng [12] proposed grey GM(1,1,t) based on the fractional-order reverse accumulative α model. Fractional calculus is applied in many fields, including image processing, control theory, fractal theory [13,14,15,16,17,18,19], etc. Wu [20] used fractional calculus to improve the GM model so that its sample would not need to be exponentially distributed and would meet the new information priority principle. Wu and his colleagues [21] successfully applied fractional accumulation to fuel production in China. Since the development of these studies, scholars have conducted in-depth research in the field of fractional-order grey models and achieved remarkable results [22,23,24,25,26,27,28,29,30]. FAGM(1,1) is the most widely used grey model at present. Few studies in the literature have provided an efficient algorithm to find the optimal order of fractional grey models. The determination of the optimal order of FAGM(1,1) has become an urgent problem. Intelligent algorithms have become the mainstay for the determination of the optimal order. In recent decades, scholars have developed various intelligent algorithms for model optimization. Among these algorithms, genetic algorithms (GAs) [31] and particle swarm optimization (PSO) [32] are relatively mature and widely used.

The contributions of this paper include the following two aspects:

(1) According to the principle of new information priority, we proposed an optimized fractional accumulative GM(1,1) model, which was applied to the prediction of cyber security situations for the first time;
(2) The combination of the genetic algorithm and particle swarm optimization was used to find the optimal order of the FAGM(1,1) model, which improved the accuracy of the model and achieved remarkable results.

## 2. Materials and Methods

### 2.1. Grey Model

The grey system theory initiated by Professor Deng, a Chinese scholar, provides a new method to solve the problem of small sample size, little information, and uncertain systems. As a new decision-making tool, this theory helps to achieve good estimations and predictions of problems in uncertain systems with limited knowledge and understanding; therefore, it rapidly gained interest among scholars in various social disciplines and scientific fields. The ubiquity of uncertainty has led to the broad development prospects of the theory. After more than 30 years of research, the grey system theory has been widely applied in agriculture, industry, society, economy, management, transportation, energy, medical care, and many other fields.

#### 2.1.1. Classic GM(1,1) Model

The main principle of GM(1,1) is generating raw data sequences into a new set of data sequences according to the models based on data sequences. Thus, a reverse calculation is carried out using the reduction method, the original data sequence is restored, and the prediction results are obtained. The data sequences are weakened by the fluctuation, and the new data are listed as follows:

The non-negative original sequence is set as follows:

(1) $$\;\;x^{\left(j\right)}=\left(x^{\left(j\right)}\left(1\right),x^{\left(j\right)}\left(2\right),\cdots x^{\left(j\right)}\left(n\right)\right)\;$$

The first-order cumulative generating sequence is set as follows:

(2) $$x^{\left(j+1\right)}=\left(x^{\left(j+1\right)}\left(1\right),x^{\left(j+1\right)}\left(2\right),\cdots x^{\left(j+1\right)}\left(n\right)\right)\;.$$

(3) $$x^{\left(j+1\right)}\left(k\right)=\sum_{i=1}^{k}x^{\left(j\right)}\left(i\right);k=i,2\cdots n.$$

The adjacent mean equal weight sequence is generated as follows:

(4) $$\left\{z^{\left(j+1\right)}\left(2\right),z^{\left(j+1\right)}\left(3\right),\cdots z^{\left(j+1\right)}\left(k\right)\right\},k=2,3\cdots n\;$$

(5) $$z^{\left(j+1\right)}\left(k\right)=0.5x^{\left(j+1\right)}\left(k-1\right)+0.5x^{\left(j+1\right)}\left(k\right),k=2,3\cdots n\;$$

The first-order one-dimensional differential equation of GM(1,1) is established as follows:

(6) $$\frac{{\mathrm{dx}}^{\left(j+1\right)}}{\mathrm{dt}}+{\mathrm{ax}}^{\left(j+1\right)}=u\;$$

where *a* represents the development coefficient, and *u* represents grey action. Band constant *Y* is generated using the cumulative data where

(7) $$B=\left[\begin{array}{cc} -z^{\left(j+1\right)}\left(2\right) & 1\\ -z^{\left(j+1\right)}\left(3\right) & 1\\ \vdots & \vdots \\ -z^{\left(j+1\right)}\left(n\right) & 1 \end{array}\right]=\left[\begin{array}{cc} -\frac{1}{2}\left(x^{\left(j+1\right)}\left(1\right)+x^{\left(j+1\right)}\left(2\right)\right) & 1\\ -\frac{1}{2}\left(x^{\left(j+1\right)}\left(2\right)+x^{\left(j+1\right)}\left(3\right)\right) & 1\\ \vdots & \vdots \\ -\frac{1}{2}\left(x^{\left(j+1\right)}\left(n-1\right)+x^{\left(j+1\right)}\left(n\right)\right) & 1 \end{array}\right],\;Y_{n}=\left[\begin{array}{c} x^{\left(j\right)}\left(2\right)\\ x^{\left(j\right)}\left(3\right)\\ \vdots \\ x^{\left(j\right)}\left(n\right) \end{array}\right]\;$$

Grey parameters are solved with the least square method as follows:

(8) $$\hat{a}={\left(B^{T}B\right)}^{-1}B^{T}Y_{n}.$$

(9) $${\hat{x}}^{\left(j+1\right)}\left(t+1\right)=\left(x^{\left(j+1\right)}\left(1\right)-\frac{u}{a}\right)e^{-\mathrm{at}}+\frac{u}{a}\;$$

The input variable of the grey model is the original sequence $\;x^{\left(j\right)}$, and the output variable is the predicted value ${\hat{x}}^{\left(j+1\right)}\left(t+1\right)$.

#### 2.1.2. Fractional Accumulative GM(1,1)

The raw data are accumulated in first order for traditional grey models. According to the research of Wu, fractional accumulative GM(1,1) is mainly used because data at different time points have different impacts on the future prediction of the model, and new information should be given more weight. The formula of fractional accumulation is as follows:

(10) $$x^{\left(r\right)}\left(k\right)=\sum_{i=1}^{k}C_{k-i+r-1}^{k-i}x^{\left(j\right)}\left(i\right)$$

(11) $$C_{k-i+r-1}^{k-i}=\frac{\left(k-i+r-1\right)\left(k-i+r-2\right)\cdots \left(r+1\right)r}{\left(k-i\right)!}\;$$

The $x^{\left(r\right)}$ sequence is accumulated using the first order.

Then, the progressive reduction operation is carried out as follows:

(12) $$x^{\left(j+1\right)}\left(k\right)=\sum_{i=1}^{k}C_{k-i+\left(1-r\right)-1}^{k-i}x^{\left(r\right)}\left(i\right)$$

(13) $$x^{\left(j\right)}\left(k\right)=x^{\left(j+1\right)}\left(k\right)-x^{\left(j+1\right)}\left(k-1\right)\;$$

The original data of the grey system are often out of order. The proper mining of internal information has always posed a significant challenge in grey systems. For better use of the information, the original data are accumulated to fully expose the integral law in the data. The original grey model presents the laws in the original discrete data through the accumulation of data. With the increase in accumulative times, the occurrences of old information increase, and the proportion becomes increasingly larger, which is contrary to the principle of giving priority to new information in grey systems. Therefore, fractional-order accumulation is proposed as a new method to solve this problem, which is used to improve the proportion of new information and the accuracy of prediction in the model. The major improvement in the fractional accumulative grey model is that the first-order accumulative operator and the first-order subtractive generation operator GM(1,1) are transformed into a fractional-order operator. Although FAGM(1,1) is similar to GM(1,1), it greatly improves the prediction accuracy of GM(1,1) and broadens the scope of the application of grey prediction models.

### 2.2. Optimization Technology

Optimization technology is an application technology based on mathematical methods to solve various engineering problems. It has been widely used in many engineering fields. Given the nonlinear, constrained, complex, and multilocal minima and modelling difficulties of practical engineering problems, the determination of new intelligent optimization methods that meet the needs of engineering practice has always been an important research direction of many disciplines.

Swarm intelligence is a general name encompassing heuristic search algorithms. This kind of algorithm is either derived from genetic research or research on group behaviour in social animals such as ants, bees, etc. In the swarm intelligence algorithm, a particle represents an individual. The particles interact with each other and follow a certain rule of motion. Through this process, the optimal solution to the problem is determined. Commonly used swarm intelligence algorithms include genetic algorithms, particle swarm optimization algorithms, etc. The optimization mechanism of such algorithms does not significantly depend on the data regarding the organizational structure of the algorithm and can widely be used in the combined optimization and calculation of functions, especially in solving some of the nonlinear, nonconvex, or nondifferentiable optimization problems of objective functions.

#### 2.2.1. Genetic Algorithm

The genetic algorithm is an optimization algorithm based on Darwin’s theory of evolution in nature. It was first proposed by Professor Holland in 1975. Genetic algorithms are mainly based on the mechanism behind natural selection, namely, the simulation of basic phenomena such as selection, crossover, and mutation in nature, the mapping of the solution of the problem with the genetic chromosome one by one, and finally the evolution of the optimal solution of the model through multiple generations of reproduction. From the perspective of the development process of artificial intelligence, genetic algorithms, evolutionary strategies, and rules together constitute the main content of biological evolutionary algorithms.

Using the coding of a genetic algorithm, the initial population is first formed. Then, based on the level of individual fitness to the environment, the evolutionary process in individual operation to achieve the survival of the fittest includes the following three basic steps:

Selection: Selection refers to the process of selecting high-quality individuals from a group and eliminating inferior individuals. It is based on fitness evaluation. High-quality individuals have a high level of fitness; they are more likely to be selected; and they will have more descendants in the next generation. Based on individual fitness values, high-quality individuals are selected from the population to enter the next step. Therefore, the higher the quality of the individuals, the stronger their ability to reproduce, and the more offspring carrying their genes.

Crossing: After high-quality individuals are selected, two individuals from this group randomly determine the crossing position to cross and form offspring. The crossing process can effectively improve the searchability of the algorithm.

Mutation: The gene on the coding string is randomly changed with a small probability, just like in natural evolution where a small number of genes are subject to gene mutation under the influence of the environment, thus enriching the diversity of the population. As children inherit the excellent characteristics of the parent generation, in theory, the children are better than the parent generation. Each iteration will eliminate individuals with low fitness values and retain those with high fitness values. In this way, the children will always cycle so the global optimal solution can be found for the fitness function.

Recently, genetic algorithms have been widely used in many practical problems, such as material identification [33], structural health monitoring [34], data mining [35], etc. With further development of this theory, the scope of its application will become more comprehensive.

#### 2.2.2. Particle Swarm Optimization

As a new intelligent computing technology, swarm intelligence optimization has received increasing attention in the scientific community. It is closely related to artificial life, evolutionary computing, and genetic algorithms. The characteristics of swarm intelligence, such as distribution, cooperation, rapidity, and robustness, provide a basis for solving complex distributed problems without centralized control and global models.

PSO is an evolutionary algorithm based on “swarm intelligence” proposed in 1995 by psychology researchers Dr. Kennedy and Dr. Elberhart, who developed their research on computational intelligence based on studying the foraging behaviour of birds. Using particle swarm optimization (PSO), the social behaviour of the biological population is simulated, and the population is driven toward the optimal direction through the sharing of individual information among the groups. This method of learning from each other’s experience among populations is not the same as the general evolutionary algorithm, which relies on the principle of natural selection and the law of evolution. The implementation method is simple and efficient. Although it was proposed for a short time, it developed rapidly. In order to improve the convergence efficiency of the particle swarm optimization algorithm, Pluhacek [36] and Tharwat [37] introduced chaos optimization into the evolution strategy of particle swarm optimization. The ergodicity of chaotic motion mitigated the problem inherent in particle optimization algorithms, namely that they easily fall into the local extremum, and it therefore improved the convergence accuracy and speed of the algorithm; Khairy [38] and Zhang [39] combined the local search strategy with the particle swarm optimization algorithm. In this model, a local search is conducted for finding the global optimal position every several generations, thus helping the algorithm jump out of the local extreme value while increasing its convergence speed. At present, it has been successfully applied to solve complex problems in system identification, pattern recognition, multi-objective optimization, system decision-making, fuzzy control, production scheduling, and other fields.

The difference between PSO algorithms and genetic algorithms is that in PSO algorithms, the selecting, crossing, and mutation of individuals do not occur; rather, each individual in the population is considered a particle without mass and volume in the multidimensional search space. These particles fly at a certain speed in the search space and dynamically adjust their own flight speed according to their flight experience and the flight experience of their peers. Each particle constantly modifies its own direction and speed by finding its optimal value and the optimal value of the population through an iterative process, thus forming a positive feedback mechanism for group optimization. In this way, with the PSO algorithm, the individual moves to a better region step by step according to the fitness of each particle to the environment and finally searches for and finds the optimal solution to the problem.

Particle swarm optimization (PSO) is a global, random search algorithm through which the chance of survival increases in each generation [40,41]. The particles include two characteristics: position and velocity. Particles update their speed and position through individual extremum ${\mathrm{pbest}}_{i}$ and group extremum ${\mathrm{gbest}}_{i}$ using the following formula:

(14) $$v_{i}=v_{i}+c_{1}\times \mathrm{rand}()\times \left({\mathrm{pbest}}_{i}-x_{i}\right)+c_{2}\times \mathrm{rand}()\times \left({\;\mathrm{gbest}}_{i}-x_{i}\right)\;$$

(15) $$x_{i}=x_{i}+v_{i}\;$$

In this formula, *i* = 1, 2, 3……*N*, *N* is the total number of particles, $v_{i}$ represents the velocity of the particle, rand() is a random number between 0 and 1, $x_{i}$ represents the current position of the particle, and $c_{1}$ and $c_{2}$ are learning factors.

It can be seen from the above that the PSO and GA algorithms have their advantages in different application scenarios.

### 2.3. The Proposed Method

In deep learning technology, large volumes of data are often used for training a model to achieve good classification or regression results; for instance, MLPNN [42] is a deep learning model that has achieved good results in the prediction of COVID-19. Due to their structure, deep neural networks have a strong expression ability compared with the traditional model, which requires more data to avoid over-fitting. Therefore, it is not advisable to use deep learning models when the sample size is small because their results are not satisfactory for small sample sizes. In this paper, we discuss the prediction of a scenario with a small sample size; therefore, a grey model is more suitable.

In GAs, previous information is lost when the population evolves through iterative processes, and they lack a high convergence speed [43]. PSOs can save the optimal information of the individual and global population because of the ability of memory. Compared with genetic algorithms, particle swarm optimization algorithms have no operators such as selection, crossover, and mutation. The structure of these algorithms is simple, and their convergence speed is relatively fast. However, particle swarm optimization algorithms easily converge prematurely and fall into a local minimum. They are weak algorithms for the optimization of integer variables [44]. This is the first study in which the advantages of GAs and PSOs are combined to optimize the fractional accumulative grey model.

**Figure 1:**
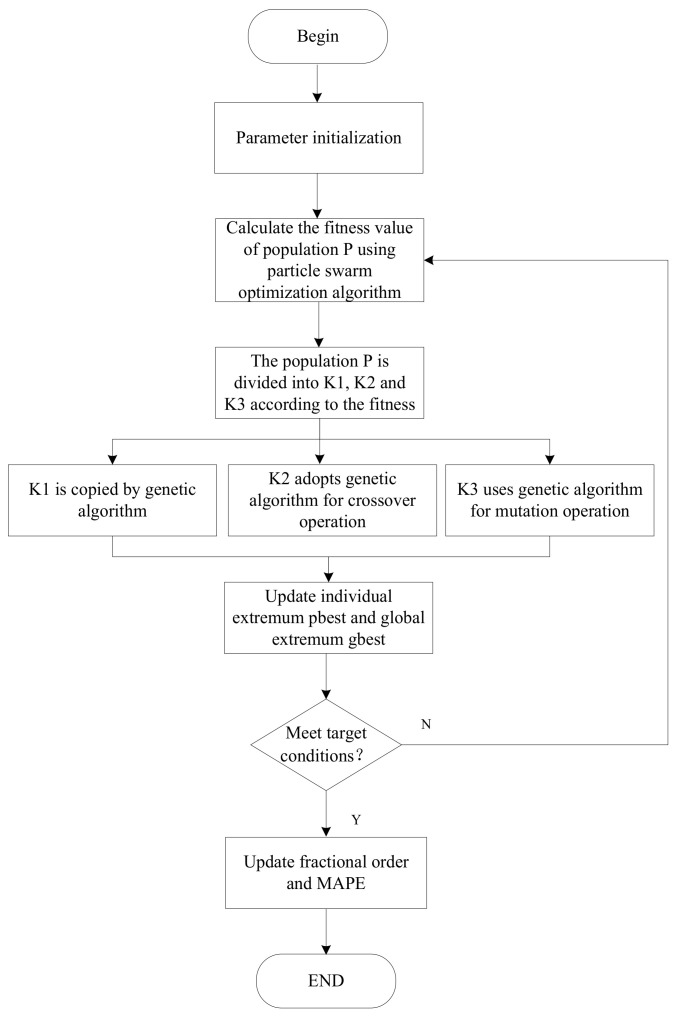
The prediction process of GAPSO-FAGM(1,1).

One of the main advantages of the proposed model is that it fits the small samples of time series and meets the principle of new information priority. Another advantage is that we used the GA-PSO algorithm to optimize the order of requirement for the achievement of better precision accuracy. The new model was applied to the prediction of security situations and had superior performance. The process of the proposed model is shown in Figure 1.

As previously mentioned, PSO and genetic algorithms have their advantages and disadvantages. A good strategy is to combine these two algorithms to form an optimizer, which can combine the advantages of the two algorithms and make up for their shortcomings. We used the GA-PSO algorithm to optimize the fractional order. The algorithm process is as follows:

Step 1: The population is initialized, with particle position X and velocity V, and the maximum number of iterations and the accuracy value of the solution are set.

Step 2: From the fitness function formula (16), it is derived that the smaller the mean absolute percentage error, the larger the corresponding fitness value, and the better the adaptability. First, a particle swarm optimization algorithm is used in population P for its optimization, and then the speed and position information of the particles are updated using Equations (14) and (15). Then, the fitness value of each particle is calculated as follows:

(16) $$\mathrm{Fitness}\;=\frac{1}{\;\mathrm{MAPE}\;},\;\mathrm{MAPE}=\frac{1}{n}\sum_{i=1}^{n}\left|\frac{y_{i}-y_{i}^{\prime }}{y_{i}}\right|$$

where $y_{i}$ denotes the actual value, $y_{i}^{\prime }$ denotes the predicted value, and *n* denotes the number of samples.

Step 3: After the population is updated, the calculated fitness values are sorted by value and divided into three parts. The P/2 particles with the highest fitness value are population K1; the P/4 particles in the middle of the ranking are population K2; and the P/4 particles with the lowest level of fitness are population K3.

Step 4: Population K1 directly replicates and enters the next generation, and population K2 performs the crossover operation of the genetic algorithm and uses the crossover probability $P_{c}$ to complete the crossover operation. The remaining population K3 uses the mutation probability $P_{m}$ for mutation operation.

Step 5: The individual extremum and group global extremum are updated.

Step 6: Steps 2 to 5 are repeated until either the objective function reaches convergence accuracy or the number of iterations reaches the set maximum number n.

## 3. Results

### 3.1. Experimental Data

The National Internet Emergency Response Center generates classified statistics on security incidents across the country and provides a basic assessment of the current state of China’s cyber security. The data samples selected in this paper are from the weekly security report published on the website of CNCERT/CC. The information and dynamic cyber security weekly report includes five types of security incidents: the number of mainframes infected with network viruses; the number of tampered networks; the number of networks implanted in backdoors; the number of counterfeit webpages on internal websites; and the number of new security vulnerabilities. The sample we selected covers 12 issues of information and dynamic cyber security weekly reports in 2016, as shown in Table 1.

The National Internet Emergency Response Center classifies the basic situation of cyber security into five levels: excellent, good, medium, poor, and dangerous. The situation values represented by each are 5, 4, 3, 2, and 1, as shown in Table 2.

**Figure 2:**
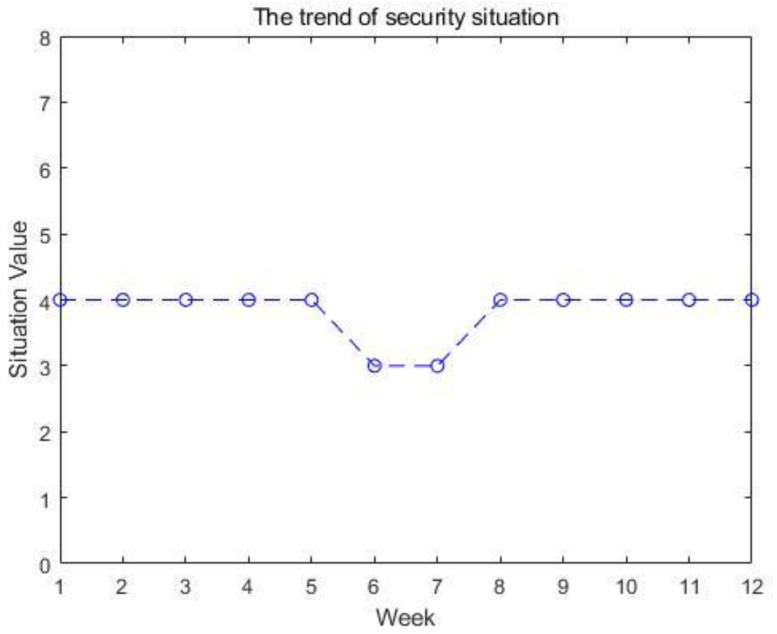
The trend of security situation.

We selected a sample comprising 12 consecutive weeks from the weekly security situation reports published on the website of CNCERT/CC to predict the cyber security situation through the proposed model. The overall trend of the cyber security situation is shown in Figure 2.

**Figure 3:**
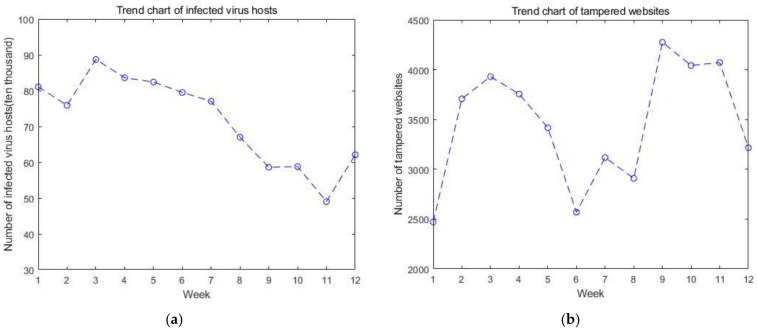

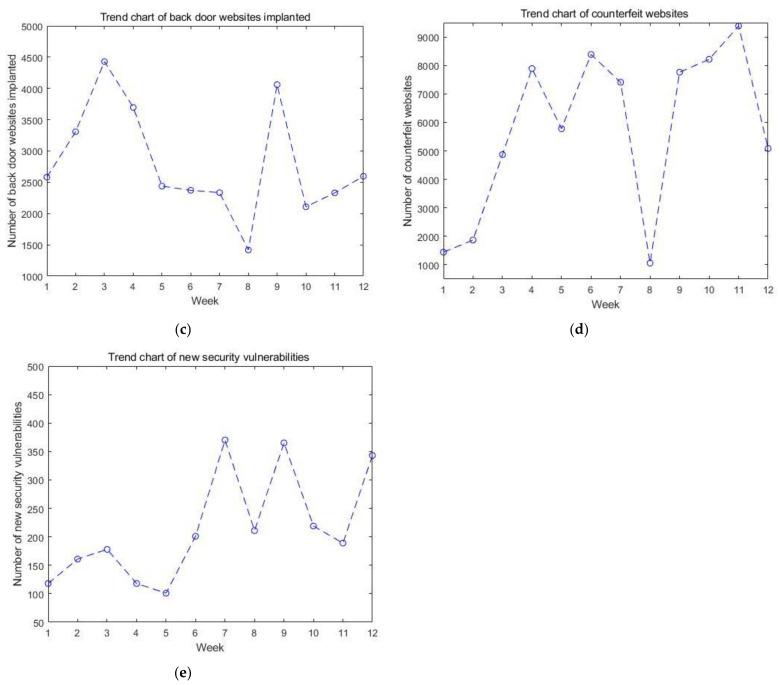
The trends of five types of incidents over time: (**a**) trend chart of infected virus hosts; (**b**) trend chart of tampered websites; (**c**) trend chart of backdoor websites implanted; (**d**) trend chart of counterfeit websites; and (**e**) trend chart of new security vulnerabilities.

Security incidents are the key factors that directly affect the situation value. The five types of incidents that affect the cyber security situation are the number of infected virus hosts, the number of tampered websites, the number of back door websites implanted, the number of counterfeit websites, and the number of new security vulnerabilities. The trends of these five types of incidents over time are in Figure 3. They have a direct impact on the results of the GM(1, n) model.

### 3.2. Experimental Environment

Hardware conditions:
CPU: Inter(R) Core(TM) i5-4590 3.30 GHZ;
Ram: 8 GB;
Hard disk: 500 GB.
Operating system: 64 bits.

### 3.3. Performance

In this paper, four evaluation indexes commonly used in predictions were selected to evaluate the prediction results: absolute error *E*, maximum absolute relative error $E_{\max}$*,* minimum absolute relative error $E_{\min\;}$, and mean absolute percentage error MAPE.

Absolute error:

(17) $$E=\left|y_{i}-y_{i}^{\prime }\right|\;$$

Maximum absolute relative error *E_max_*:

(18) $$E_{\max}=\left|\frac{y_{i}-y_{i}^{\prime }}{y_{i}}\right|\;$$

Minimum absolute relative error *E_min_*:

(19) $$\;E_{\min\;}=\left|\frac{y_{i}-y_{i}^{\prime }}{y_{i}}\right|\;$$

Mean absolute percentage error:

(20) $$\mathrm{MAPE}=\frac{1}{n}\sum_{i=1}^{n}\left|\frac{y_{i}-y_{i}^{\prime }}{y_{i}}\right|$$

**Figure 4:**
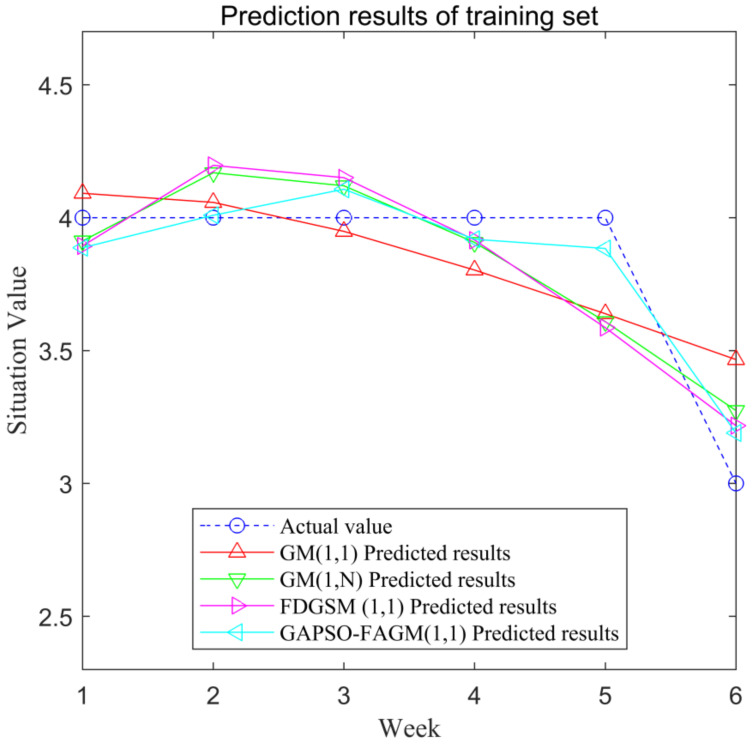
Prediction results of the training set.

**Figure 5:**
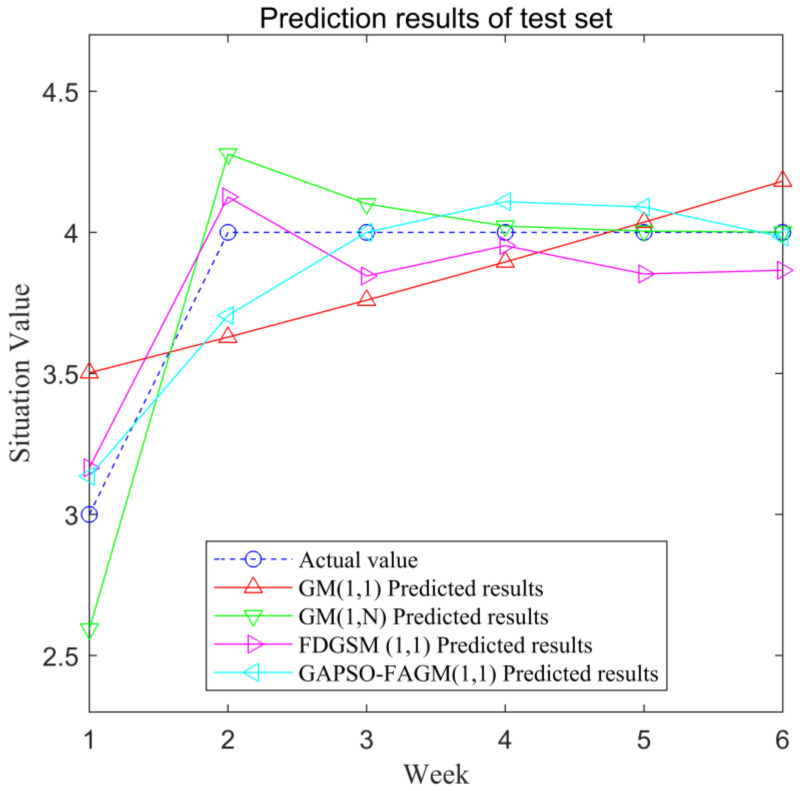
Prediction results of the test set.

We divided the 12 groups of data into 2 groups, due to the small sample size: the first 6 groups of data were used as training sets, and the remaining 6 groups of data were used as test sets. The prediction results of the training set are shown in Figure 4. The prediction results of the test set are shown in Figure 5.

The predicted values of the training set in the four models and their relative errors, as shown in Table 3.

The predicted values of the test set with the four models and their relative errors, as shown in Table 4.

**Figure 6:**
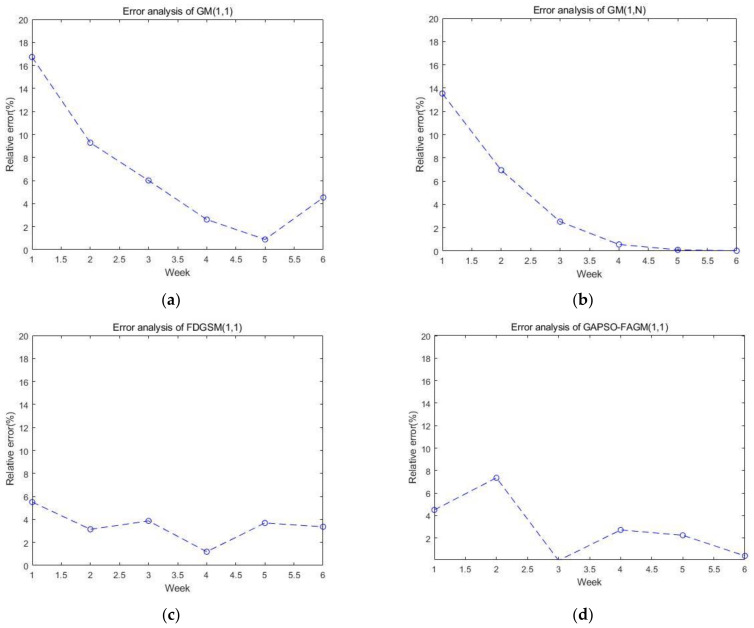
The error analysis of the test set using the four models: (**a**) error analysis of GM(1,1); (**b**) error analysis of GM(1,N); (**c**) error analysis of FDGSM(1,1); and (**d**) error analysis of GAPSO-FAGM(1,1).

The diagrams of the relative errors for the test set using the four models are presented in Figure 6. We can see that the average relative error of FAGM(1,1) optimized by the GA and PSO algorithms is smaller than others; however, there is still room for improvement in the stability of prediction.

The *MAPE* values of the training set using four models are shown in Table 5. The *MAPE* values of GM(1, 1), GM(1,n), FDGSM(1,1) [30], and GAPSO-FAGM(1,1) are 5.75%, 5.11%, 5.17%, and 2.83%, respectively. It can be seen that the *MAPE* of GAPSO-GM(1,1) is the smallest among the four models.

The *MAPE* values of the test set using the four models are shown in Table 6. The *MAPE* values of GM(1,1), GM(1,n), FDGSM(1,1), and GAPSO-FAGM(1,1) are 6.68%, 3.95%, 3.46%, and 2.87%, respectively. It can be seen that the *MAPE* of GAPSO-GM(1,1) is the smallest among the four models.

## 4. Discussion

Compared with currently popular methods such as machine learning, grey models have achieved remarkable advantages in cost prediction in cases with little information and small samples; therefore, researchers have focused on these models when dealing with small samples. The advent of the fractional-order cumulative grey model makes a big leap forward in the research of grey models. Fractional-order accumulation gives more weight and higher priority to new information, thus making grey models more realistic in prediction with higher accuracy. However, fractional-order cumulative grey models have always been ineffective in solving the optimal order. In this paper, a new fractional grey model is proposed. The optimizer (GA-PSO) formed by combining the advantages of the GA and PSO algorithms was used to adjust its fractional order, which fills the gap in research regarding the order optimization of fractional grey models. Based on the GM(1,1) model, we used fractional order instead of integer order to take the weight of information into account. The GA-PSO algorithm was used to determine the order of FAGM(1,1), making the prediction result optimal. Finally, the proposed model was applied to predict the cyber security situation. A comparison with the outcome of traditional GM(1,1), FDGSM(1,1), and GM(1,N) revealed that fractional-order accumulation and the order optimization of GA-PSO play important roles in prediction accuracy and model stability.

## 5. Conclusions

The research on the prediction of network security situations based on small sample sizes is of great significance. Grey models have made remarkable achievements in small sample prediction, and fractional cumulative grey models present a relatively successful improvement in grey models. This paper mainly focused on the intelligent optimization of fractional grey models. The GA and PSO algorithms have achieved good results in swarm intelligence optimization, but they have their disadvantages; the combination of these two algorithms can eliminate some of their defects. Therefore, in this paper, we proposed to determine the optimal fractional order based on a fractional grey model using a combination of the GA and PSO algorithms, so as to establish a fractional grey model based on intelligent optimization. Our experiments revealed that this model achieved good results and more accurately predicted the cyber security situation. Further studies are needed to apply the model to other areas. In future work, researchers might explore how to improve the stability of prediction. In addition, as this paper mainly focused on short-term prediction, the combination of this model with deep-learning-based algorithms for medium and long-term prediction will be the focus of our next study.

## Figures and Tables

**Table 1:** Data sample.

SN	Cyber Security Situation Level	Number of Infected Virus Hosts	Number of Tampered Websites	Number of Back Door Websites Implanted	Number of Counterfeit Websites	Number of New Security Vulnerabilities
1.	4	81.05	2471	2581	1444	118
2.	4	75.9	3708	3309	1870	161
3.	4	88.7	3930	4430	4870	178
4.	4	83.6	3756	3697	7889	118
5.	4	82.39	3417	2438	5778	101
6.	3	79.5	2569	2372	8384	201
7.	3	77.04	3118	2334	7410	370
8.	4	67	2909	1418	1061	211
9.	4	58.6	4275	4061	7761	365
10.	4	58.8	4042	2108	8212	219
11.	4	49.03	4072	2330	9384	189
12.	4	62.1	3217	2596	5082	343

**Table 2:** Levels of cyber security situation.

Excellent	Good	Medium	Poor	Dangerous
5	4	3	2	1

**Table 3:** The predicted values of the training set in the four models and their relative errors.

Sn.	Actual Value	GM(1,1)		GM(1,N)		FDGSM(1,1)		GAPSO-FAGM(1,1)	
		Values	Relative Error (%)	Values	Relative Error (%)	Values	Relative Error (%)	Values	Relative Error (%)
1	4	4.0917	2.2937	3.9123	2.1922	3.8938	2.6559	3.887	2.825
2	4	4.0575	1.4376	4.1690	4.2242	4.1967	4.9170	4.0090	0.225
3	4	3.9484	1.2891	4.1199	2.9973	4.1505	3.7626	4.1070	2.675
4	4	3.8028	4.9293	3.9067	2.3337	3.9159	2.1018	3.9187	2.0325
5	4	3.6387	9.0314	3.6089	9.7770	3.5861	10.3467	3.8840	2.9
6	3	3.4661	15.5361	3.2743	9.1436	3.2173	7.2422	3.1897	6.3233

**Table 4:** The predicted values of the test set with the four models and their relative errors.

Sn.	Actual Value	GM(1,1)		GM(1,N)		FDGSM(1,1)		GAPSO-FAGM(1,1)	
		Values	Relative Error (%)	Values	Relative Error (%)	Values	Relative Error (%)	Values	Relative Error (%)
1	3	3.5020	16.73	2.5940	13.53	3.1654	5.51	3.1350	4.50
2	4	3.6284	9.29	4.2778	6.94	4.1255	3.14	3.7055	7.36
3	4	3.7594	6.01	4.1010	2.52	3.8454	3.87	4.0001	0.0024
4	4	3.8951	2.62	4.0222	0.56	3.9527	1.19	4.1087	2.7185
5	4	4.0358	0.89	4.0042	0.10	3.8526	3.69	4.0897	2.2435
6	4	4.1815	4.53	4.0010	0.02	3.8656	3.36	3.9835	0.4132

**Table 5:** *MAPE* of the training set using the four models.

GM(1,1)	GM(1,n)	FDGSM(1,1)	GAPSO-GM(1,1)
5.75%	5.11%	5.17%	2.83%

**Table 6:** *MAPE* of the test set using the four models.

GM(1,1)	GM(1,n)	FDGSM(1,1)	GAPSO-GM(1,1)
6.68%	3.95%	3.46%	2.87%

## Data Availability

The raw data are available from https://www.cert.org.cn/, accessed on 18 December 2021.
